# A Simple SERS-Based Trace Sensing Platform Enabled by AuNPs-Analyte/AuNPs Double-Decker Structure on Wax-Coated Hydrophobic Surface

**DOI:** 10.3389/fchem.2018.00482

**Published:** 2018-10-16

**Authors:** Huixiang Wu, Yi Luo, Yikun Huang, Qiuchen Dong, Changjun Hou, Danqun Huo, Jing Zhao, Yu Lei

**Affiliations:** ^1^Key Laboratory for Biorheological Science and Technology of Ministry of Education, State and Local Joint Engineering Laboratory for Vascular Implants, Bioengineering College of Chongqing University, Chongqing, China; ^2^Department of Chemical and Biomolecular Engineering, University of Connecticut, Storrs, CT, United States; ^3^Department of Chemistry, University of Connecticut, Storrs, CT, United States; ^4^Department of Biomedical Engineering, University of Connecticut, Storrs, CT, United States

**Keywords:** wax, gold nanoparticles, surface enhanced Raman scattering, methyl parathion, melamine

## Abstract

In this work, a simple and versatile SERS sensing platform enabled by AuNPs-analyte/AuNPs double-decker structure on wax-coated hydrophobic surface was developed using a portable Raman spectrometer. Wax-coated silicon wafer served as a hydrophobic surface to induce both aggregation and concentration of aqueous phase AuNPs mixed with analyte of interest. After drying, another layer of AuNPs was drop-cast onto the layer of AuNPs-analyte on the substrate to form double-decker structure, thus introducing more “hot spots” to further enhance the Raman signal. To validate the sensing platform, methyl parathion (pesticide), and melamine (a nitrogen-enrich compound illegally added to food products to increase their apparent protein content) were employed as two model compounds for trace sensing demonstration. The as-fabricated sensor showed high reproducibility and sensitivity toward both methyl parathion and melamine detection with the limit of detection at the nanomolar and sub-nanomolar concentration level, respectively. In addition, remarkable recoveries for methyl parathion spiked into lake water samples were obtained, while reasonably good recoveries for melamine spiked into milk samples were achieved. These results demonstrate that the as-developed SERS sensing platform holds great promise in detecting trace amount of hazardous chemicals for food safety and environment protection.

## Introduction

Surface enhanced Raman scattering (SERS) has been drawing increasing attention in the field of analytical chemistry and life science since its first introduction about 40 years ago (Fleischmann et al., 1974; Albrecht and Creighton, 1977; Jeanmaire and Duyne, 1977; Freeman et al., 1995; Wang et al., 2013; Zhou et al., 2016). SERS enhancement derives from giant electromagnetic field enhancement enabled by localized surface plasmon resonance (LSPR) “hot spots,” which are typically formed at small gaps (usually < 10 nm) between noble metal nanoparticles, with an enhancement factor up to 10^12^ (Kleinman et al., 2013). Compared with traditional bulky instruments, for instance gas chromatography (Vesely et al., 2003), liquid chromatography (Hogard et al., 2017), high-performance liquid chromatography (Özyürek et al., 2012) etc., SERS based detection can be realized using a portable Raman spectrometer, which displays several advantages, such as portability, easy accessibility, cost effectiveness, and rapid analysis. These advantages endow its potential use in point-of-care and in-field trace sensing applications.

Gold nanoparticles (AuNPs) have been widely employed as SERS substrates because they offer very strong Raman enhancing effect (Lou et al., 2011; Li J. J. et al., 2015; Jiang et al., 2017). Furthermore, excellent size and shape tunability of AuNPs make it possible to optimize the enhancement factor for desired SERS detection. To date, scores of reports have been published on AuNPs-based SERS for the detection and discrimination of DNA (Lim et al., 2010), anticancer drugs (Ilkhani et al., 2016; Kurzatkowska et al., 2017), amino acid (Schwartzberg et al., 2004), melamine (Lee et al., 2007; He et al., 2011; Kim et al., 2012; Giovannozzi et al., 2014; Rajapandiyan et al., 2015), biothiols (Liu et al., 2017), various pesticides (Nguyen et al., 2014; Alsammarraie and Lin, 2017; Cao et al., 2017; Fortuni et al., 2017; Jiang et al., 2017; Tan et al., 2017), and other hazardous chemicals (Li et al., 2011). For example, Giovannozzi et al. (2014) presented a simple AuNPs based SERS system in aqueous solution for melamine detection, in which colloidal AuNPs displayed satisfactory SERS enhancement with a limit of detection of 1.35 μM melamine. Although acceptable sensitivity and reproducibility have been obtained for melamine detection in aqueous solution, more sensitive detection for hazardous chemicals is still highly demanded, especially for the detection of highly toxic compounds such as pesticides.

Generally, SERS detection of analyte on solid state substrates is much more sensitive than detection of analyte in aqueous solutions due to the formation of more “hot spots” among adjacent nanoparticles. A host of SERS substrates were fabricated based on highly homogeneous noble metal nanostructures (e.g., Au nanorod arrays) to improve the reproducibility (Kim et al., 2012; Peng et al., 2013; Wallace et al., 2014; Yu et al., 2015; Wang et al., 2016). Although favorable repeatability and excellent sensitivity toward targets can be achieved by using those SERS substrates, relatively high-cost associated with complicated fabricating processes could potentially hinder their wide applications. Alternatively, the use of hydrophobic substrate can also enhance sensitivity and reproducibility of the SERS based sensor system. In this scheme, small volume of aqueous samples were dropped on a hydrophobic substrate and allowed to evaporate. The evaporation process created a higher number of “hot spots” in confined area and better uniformity of nanomaterials distribution. Accordingly, many efforts have been made to produce and employ hydrophobic SERS substrates for enhanced SERS sensing (Li et al., 2014; Wallace et al., 2014; Cheung et al., 2016; Jayram et al., 2016). However, a simple method to fabricate hydrophobic substrate is still highly demanded.

**Figure 1:**
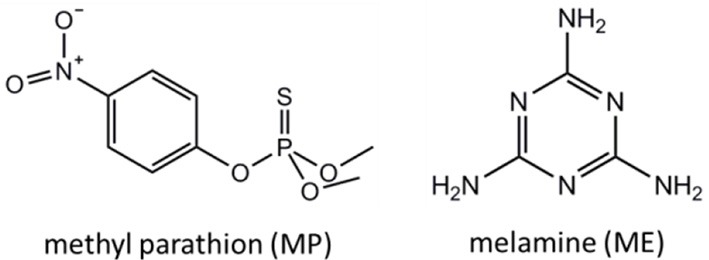
Chemical structures of methyl parathion and melamine, respectively.

In this work, we present a simple and versatile SERS sensing platform enabled by AuNPs-analyte/AuNPs double-decker structure on wax-coated hydrophobic surface using a portable Raman spectrometer. The simplicity, cost-effectiveness, and easiness to handle in coating are three greatest merits of wax serving as a hydrophobic material, compared to other hydrophobic materials in SERS applications. Wax-coated silicon wafer served as a hydrophobic surface to induce both aggregation and concentration of AuNPs mixed with analyte of interest. After drying, another layer AuNPs of aqueous phase was drop-cast onto the layer of AuNPs-analyte on the substrate to form a double-decker structure, inducing more “hot spots” to further enhance the Raman signal. Specifically, AuNPs with diameters of 60 and 130 nm were employed for SERS detection of two model compounds, methyl parathion and melamine (chemical structure shown in Figure 1) respectively, to demonstrate the feasibility of the developed SERS sensing platform for environment and food safety monitoring. The SERS sensor showed high sensitivity with a detection limit at nanomolar/sub-nanomolar level toward the two target molecules, with satisfactory reproducibility. In addition, the sensor was able to detect both methyl parathion spiked into lake water with remarkable recovery and melamine spiked into milk with reasonably good recovery. These results suggest that this simple, low cost, and versatile SERS sensing platform can offer a promising strategy for ultrasensitive detection of hazardous chemicals in real samples.

## Materials and methods

### Chemicals and reagents

Chloroauric acid (HAuCl_4_·4H_2_O, ≥99.9% trace metals basis), hydroxylamine hydrochloride (NH_2_OH·HCl), methyl parathion (C_8_H_10_NO_5_PS, denoted as MP) and melamine (C_3_H_6_N_6_, denoted as Me) were acquired from Sigma-Aldrich. Sodium citrate (Na_3_C_6_H_5_O_7_·2H_2_O) was purchased from Fisher Scientific. All of the chemicals were used without any purification.

### Instrument and apparatus

UV-vis spectra of colloidal AuNPs were recorded on a UV-spectrometer (Cary 60, Agilent Technologies). Transmission electron microscopy (TEM) images and scanning electron microscopy (SEM) images were acquired using FEI Tecnai G2 Spirit BioTWIN and FEI Nova NanoSEM 450, respectively. A portable Raman spectrometer (QE Pro, Ocean Optics), coupled with a 785 nm laser (operated at 35 mW), was used to collect the Raman spectra. For each Ramanmeasurement, the spectrumwas integrated for 5 s.

### Preparation of AuNPs

Sixty nanometers of AuNPs were prepared using Frens' method (Frens, 1973). Briefly, 1.06 mg of chloroauric acid aqueous solution (0.0254 M) was added into a 250 mL round-bottomed flask loaded with 99 mL distilled water and heated to boil. After that, 1.0 mL of freshly prepared sodium citrate aqueous solution (0.0388 M) was added into the mixture with stirring and kept boiling for another 20 min. Finally, 60 nm AuNPs were obtained after turning off the heating and cooling to room temperature with stirring.

A seed mediated method was used to synthesize 130 nm AuNPs according to previous report (Tian et al., 2013). In detail, 4 mL of as-prepared 60 nm AuNPs, 900 μL of freshly prepared sodium citrate aqueous solution (0.0388 M) and 52 mL deionized water were successively loaded into the flask with stirring for 5 min. To the above solution, 0.88 mL of aqueous chloroauric acid solution (0.0254 M) was quickly added and kept stirring for another 5 min. After that, 700 μL of freshly prepared hydroxylamine hydrochloride aqueous solution (0.0101 M) was injected into above mixture solution twice and the reaction solution was incubated for 2 h to yield AuNPs with an average diameter of about 130 nm.

### Preparation of methyl parathion and melamine samples

Methyl parathion stock solution (0.01 M) was prepared by adding 0.0263 g of methyl parathion into 10 mL absolute ethanol, while melamine stock solution (0.01 M) was prepared by adding 0.0126 g of melamine into 10 mL of deionized water. Methyl parathion ethanol solutions and melamine aqueous solutions with certain concentrations were obtained by diluting the stock solutions using ethanol and deionized water correspondingly.

### Preparation of spiked real samples

Lake water sample was collected from Swan Lake at the University of Connecticut and filtered through an ordinary filter paper to remove any large solid particles. Methyl parathion spiked lake water samples were then prepared by quantitatively adding appropriate amount of methyl parathion stock solution into lake water samples and finally subject to SERS detection in recovery studies.

Milk samples (purchased from a local grocery store) were first spiked with certain concentrations of melamine and then pretreated according to a previous report with some modifications for recovery studies (Hu et al., 2015). Briefly, milk samples (0.5 mL) spiked with different concentration of melamine were added into 4.5 mL methanol and shaken for 1 min, followed by sonication for 10 min. And then, the mixtures were centrifuged for 10 min at 12,000 rpm. Supernatants were collected and subject to SERS detection in recovery studies.

### SERS measurement procedure

**Figure 2:**
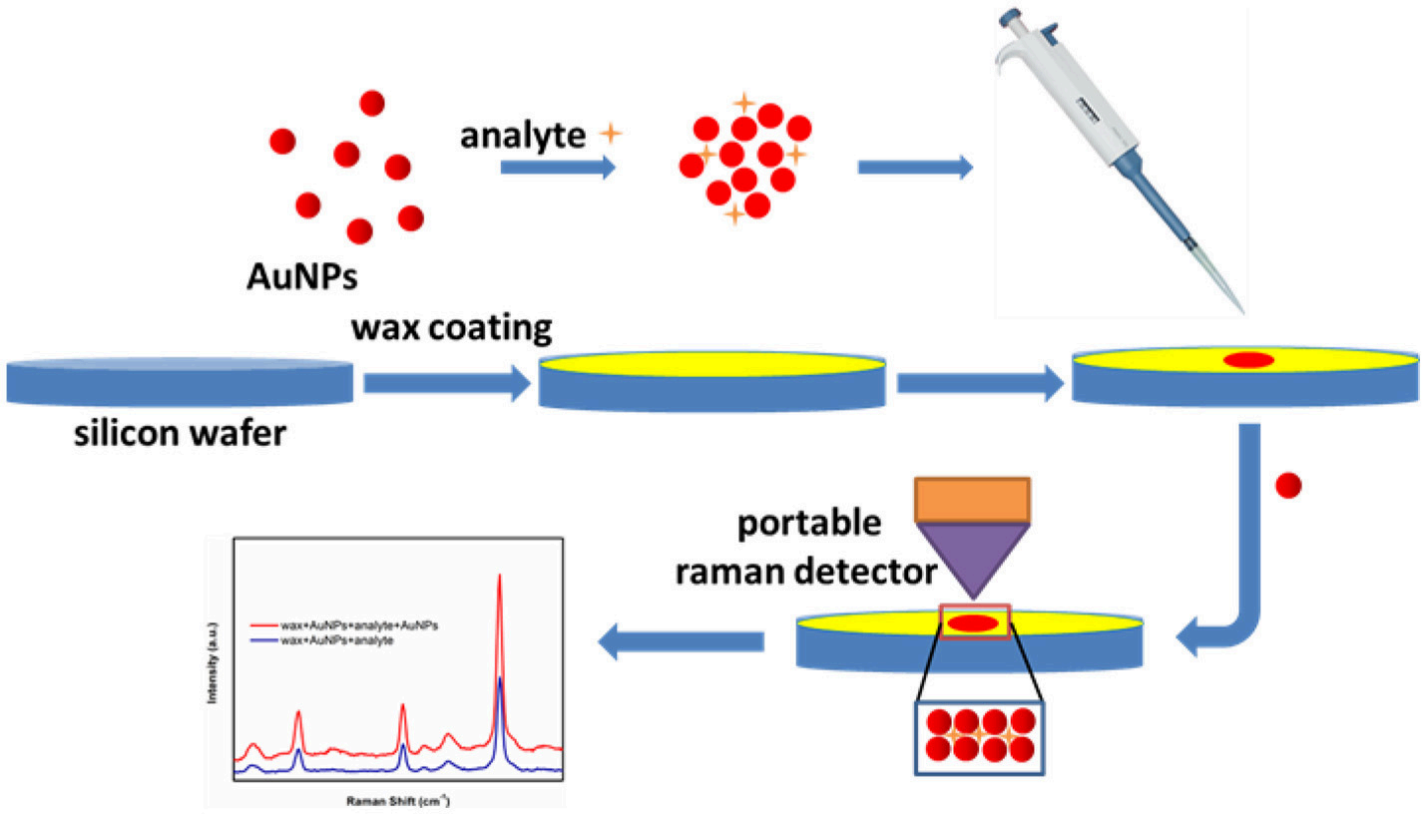
Schematic of the SERS-based trace sensing platform enabled by AuNPs-analyte/AuNPs double-decker structure on wax-coated hydrophobic surface.

The typical SERS sensing procedure developed in this study is presented in Figure 2 and briefly described below. Commercial wax was first heated and melted into liquid state in a crucible. Then liquid wax was poured onto a silicon wafer, immediately followed by spreading using a glass rod before its solidification, thus generating a thin layer of wax to offer an excellent hydrophobic surface. Next, 5 μL methyl parathion samples or melamine samples were added into 45 μL AuNPs (with certain particle size) aqueous solutions and incubated for 5 min to form AuNPs-analyte mixture solution. Next, 5 μL AuNPs-analyte mixture solution were dropped onto the wax-coated surface and left to dry in ambient conditions. After drying, 5 μL of AuNPs solution were subsequently dropped onto the dried spot and allowed to dry again. Finally, Raman scattering spectra were recorded from the dried spots using a portable Raman spectrometer.

## Results and discussion

### Characterization of AuNPs

**Figure 3:**
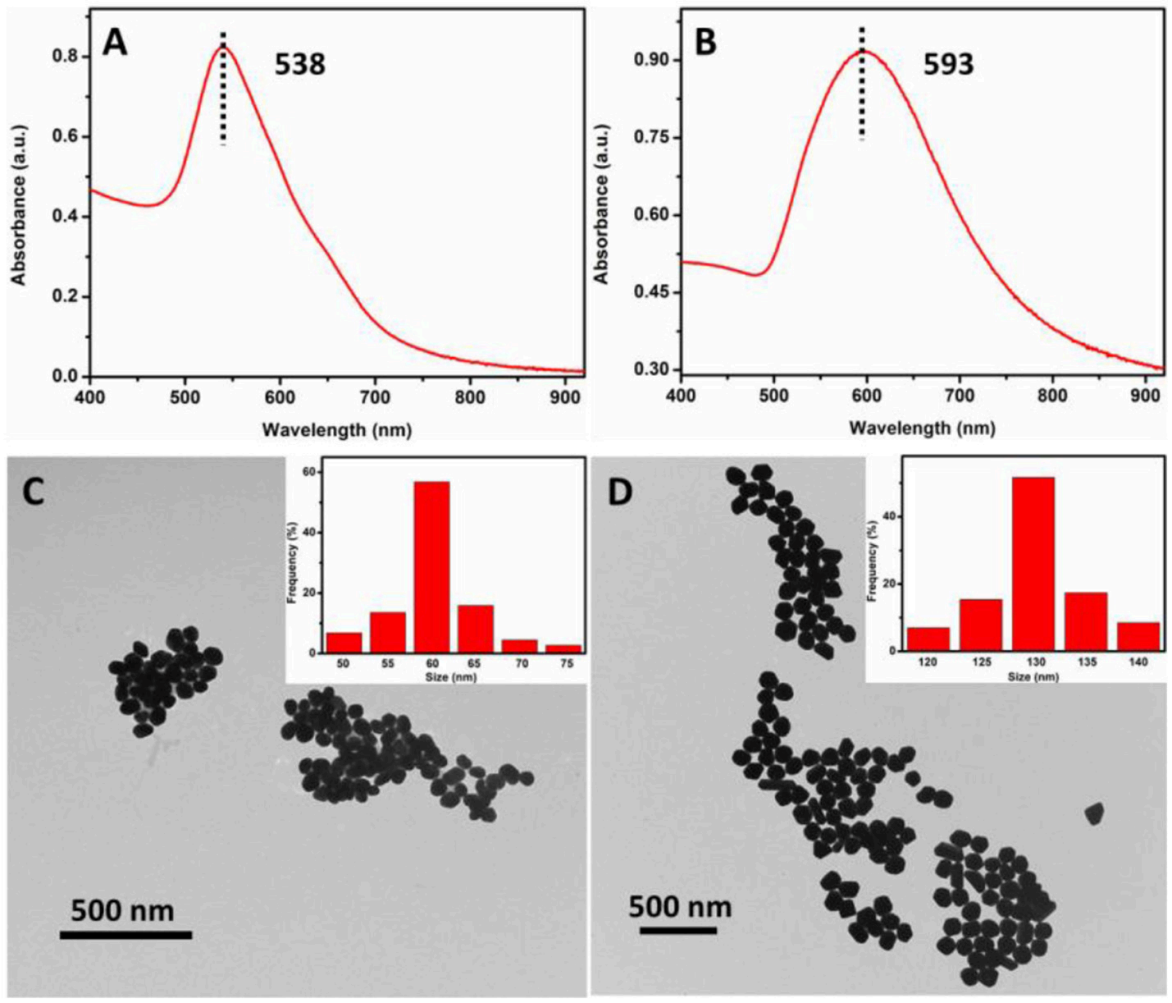
UV-vis spectra **(A,C)** and TEM images **(B,D)** of the as-prepared 60 and 130 nm AuNPs, respectively.

UV-vis spectroscopy and TEM were employed to determine the absorption spectrum and morphology of the AuNPs. As shown in Figure 3A, UV-vis spectrum of the as-prepared 60 nm AuNPs suspension has a narrow peak at 538 nm, indicating that AuNPs were monodispersed. To further examine the morphology of the 60 nm AuNPs, TEM image was collected. From Figure 3C and its inset, one can see that the AuNPs show good size-uniformity with an average diameter of about 60 nm. Similarly, Figure 3B shows the UV-vis spectrum of the as-prepared 130 nm AuNPs synthesized. A relatively broad peak was observed with a peak wavelength of 593 nm, suggesting lower size uniformity than that of the 60 nm AuNPs. From the TEM image in Figure 3D, the AuNPs have an average diameter of about 130 nm, with some variation in their shapes, such as sphere, polyhedron and rod-like. The concentration of the as-prepared 60 and 130 nm AuNPs was calculated to be 0.0250 and 0.0058 nM, respectively, according to its UV-vis spectrum and a reported method (Haiss et al., 2007). And the as-prepared 60 and 130 nm AuNPs were then utilized for subsequent SERS studies.

### Hydrophobicity of wax-coated silicon wafer

**Figure 4:**
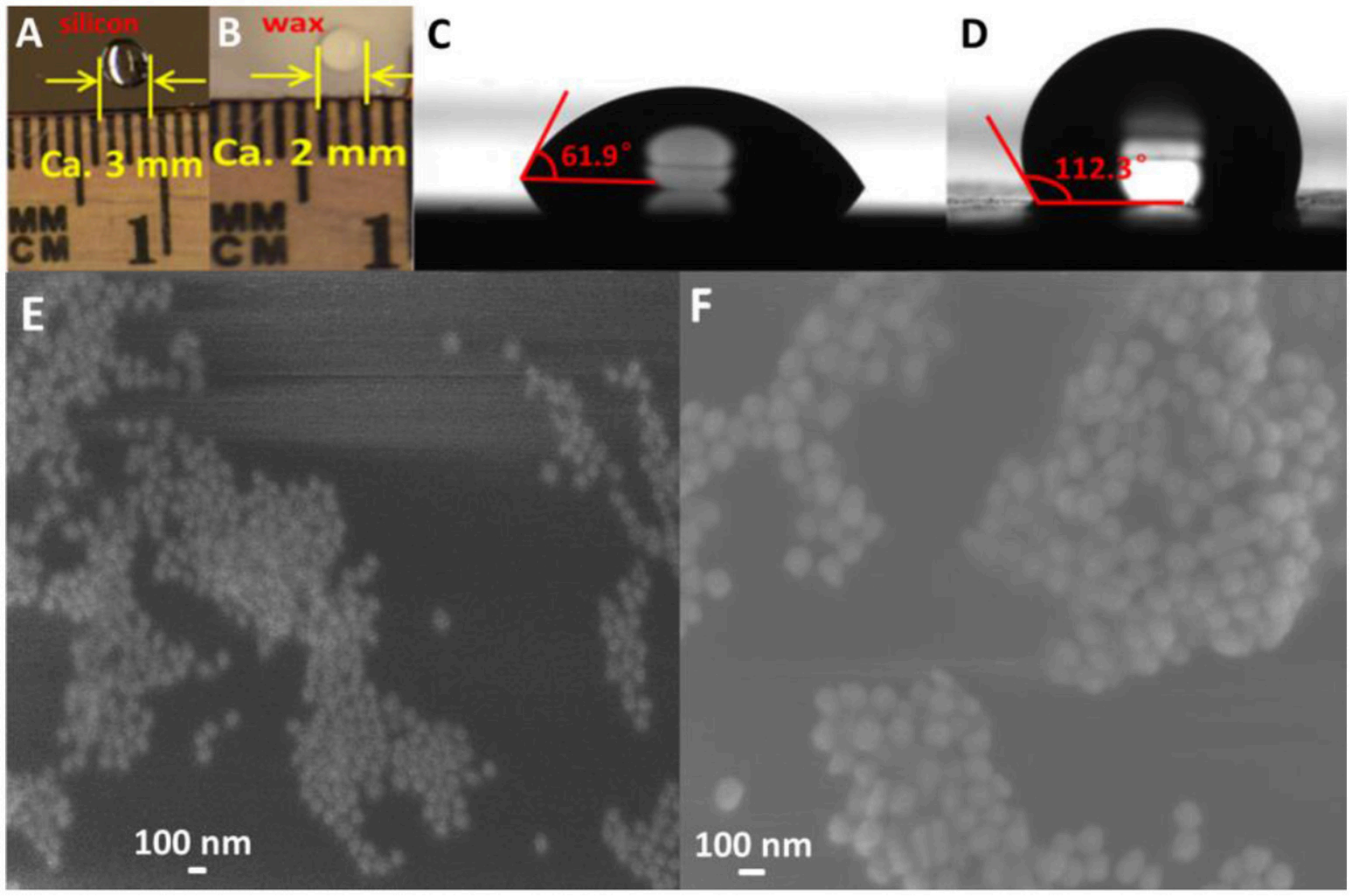
Optical pictures of water droplet on silicon wafer **(A)** and wax-coated silicon wafer **(B)**; Contact angles of water drop and silicon wafer **(C)** and wax-coated silicon wafer **(D)**; SEM images of AuNPs-methyl parathion/AuNPs **(E)** and AuNPs-melamine/AuNPs **(F)**.

In this study, hydrophobicity of the substrate plays important roles in both the reproducibility and the sensitivity of SERS sensor. Figures 4A,B show the photograph of a 5 μL water droplet on the silicon wafer and a thin layer of wax-coated silicon wafer, respectively. The diameter (ca. 2 mm) of the water droplet on the wax-coated silicon wafer was obviously smaller than that (ca. 3 mm) on the bare silicon wafer, suggesting good hydrophobicity of the simple wax coating. To further study the hydrophobicity of wax-coated silicon wafer, the liquid-solid contact angle test was carried out. As shown in Figure 4C, a contact angle of 61.9° was observed for water droplet on bare silicon wafer. In comparison, the contact angle for wax-coated silicon wafer was measured to be 112.3° according to Figure 4D, confirming the much higher hydrophobicity of wax-coated silicon wafer than the bare one. To examine the morphology of the AuNPs layer on the substrate, 60 nm AuNPs were first added on the wax-coated silicon wafer. Due to the hydrophobicity of the substrate, the AuNPs typically formed small aggregates on the substrate (data not shown). After the addition of another layer of AuNPs, a dense AuNPs aggregates formed as seen from Figure 4E. One can see that a great number of gaps formed in the AuNPs aggregates, which serve as “hot spots” for SERS detection. Similar phenomena were observed for 130 nm AuNPs on substrates (Figure 4F). The data suggest that the wax coating on silicon wafer induced aggregation of the AuNPs, and the additional layer of AuNPs introduced more “hot spots,” thus enhancing the sensitivity in subsequent SERS measurement.

### Raman spectra intensities of targets performed on various substrates

**Figure 5:**
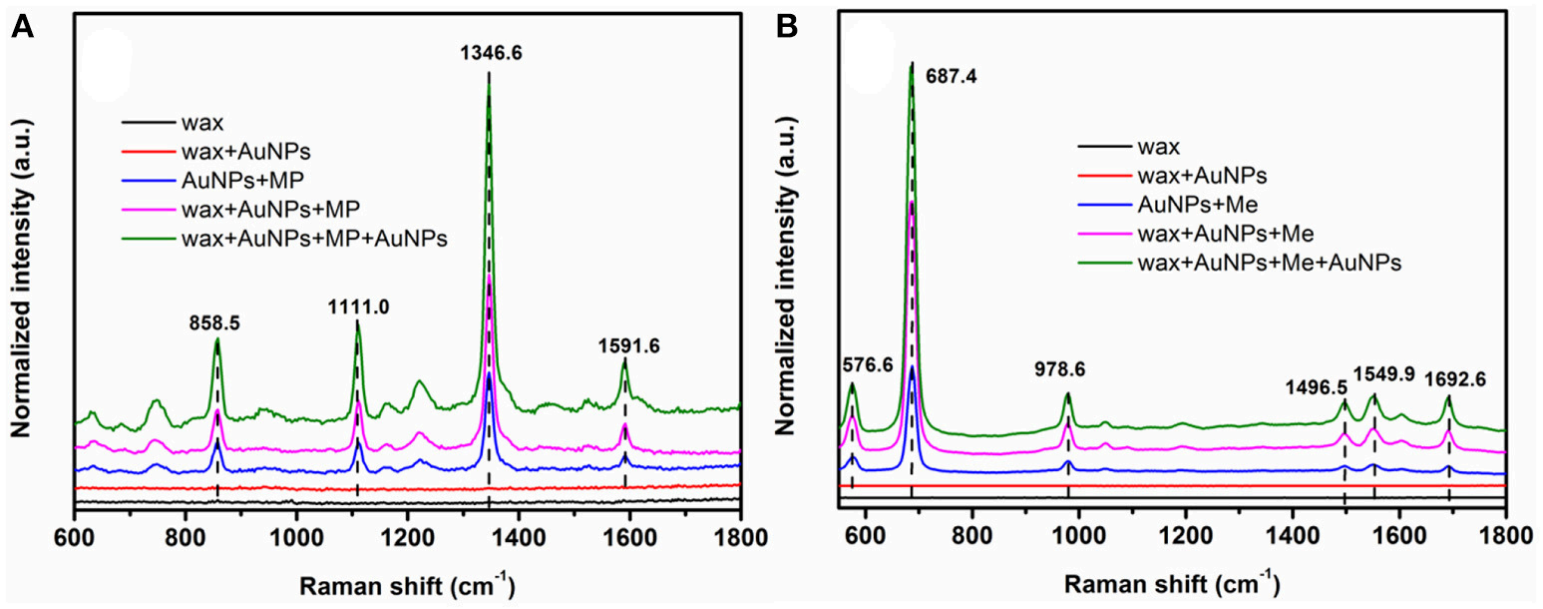
**(A)** Raman scattering spectra of wax (black), wax coated with 60 nm AuNPs (red), mixture of methyl parathion (10^−4^ M) and 60 nm AuNPs deposited on silicon wafer (blue), methyl parathion (10^−4^ M) and AuNPs deposited on wax-coated silicon wafer (pink), and methyl parathion (10^−4^ M) and AuNPs deposited on wax-coated silicon wafer with an additional layer of AuNPs (green). **(B)** Raman scattering spectra of wax (black), wax coated with 130 nm AuNPs (red), mixture of melamine (10^−5^ M) and 130 nm AuNPs deposited on silicon wafer (blue), melamine (10^−5^ M) and AuNPs deposited on wax-coated silicon wafer (pink), and melamine (10^−5^ M) and AuNPs deposited on wax-coated silicon wafer with an additional layer of AuNPs (green).

Better hydrophobicity of the substrate, on one hand, can shrink the AuNPs-analyte droplet during evaporation and lead to higher target concentration and more “hot spots” in the probe spot, enhancing the sensitivity. On the other hand, better hydrophobicity of the substrate improves the uniformity of the distribution of AuNP-analyte complexes on the substrate after drying, thus favoring better reproducibility of SERS sensing. As shown in Figure 5, Raman scattering peak intensities of the analyte on wax-coated silicon wafer were much stronger than that on the bare silicon wafer, for both of methyl parathion and melamine, at the same analyte concentration. In addition, no obvious Raman scattering peak was observed for wax with or without AuNPs coating (black and red lines in Figures 5A,B), indicating that wax did not interfere with the Raman measurement. Four strong peaks appeared at 858.5 cm^−1^, 1111.0, 1346.6, and 1591.6 cm^−1^ in the Raman spectra collected from methyl parathion on 60 nm AuNPs on silicon wafer (blue line in Figure 5A). They can be assigned to the stretching vibration of P-O and C-N, bending vibration of C-H and phenyl stretching, respectively, in accordance with previous report (Li Z. et al., 2015). Peaks at identical positions with stronger intensities were observed in the Raman spectra for AuNPs-methyl parathion on wax-coated silicon wafer (pink line in Figure 5A). Similar phenomena were observed in Figure 5B for complex of AuNPs (130 nm) and melamine on silicon wafer. Specifically for melamine detection, the strongest Raman peak at 687.4 cm^−1^ was attributed to ring breathing. Peaks at 576.6, 978.6, 1496.5, 1549.9, and 1692.6 cm^−1^ were comprehensively ascribed to vibrations of C-N and N-H (Mircescu et al., 2012). All these distinct assignments were listed in Table 1. Clearly, intensities of these peaks of AuNPs-melamine on wax-coated silicon wafer were much stronger than those on bare silicon wafer.

**Table 1:** Raman scattering peaks assignment for methyl parathion and melamine (Mircescu et al., 2012; Li Z. et al., 2015).

**Species**	**Observed peaks(cm ^−1^)**	**Vibrational description**
Methyl parathion (MP)	858.5	ν(P–O)
	1111.0	ν(C–N)
	1346.6	δ(C–H)
	1591.6	Phenyl stretching
Melamine (Me)	576.6	δ(NCN)+τ(NH_2_)
	687.4	Ring breathing
	978.6	δ(CNC)+τ(NCN)
	1496.5	δ(NCN)+ω(NH_2_)
	1549.9	ν(CN)+δ(NH_2_)
	1692.6	δ(NH_2_)

*ω, wagging vibration; δ, bending vibration; ν, stretching vibration; τ, twisting vibration*.

“Hot spots” have been demonstrated to be extremely important for SERS based sensing (Camden et al., 2008; Kubackova et al., 2015; Zhang et al., 2017). In order to further improve the sensitivity of the sensor, another AuNPs layer was coated onto the AuNPs-analytes on wax, forming a spot of AuNPs-analytes/AuNPs double-decker structure on wax. The additional AuNPs coating can substantially increase the amount of small gaps between AuNPs-analytes and AuNPs, resulting in a sandwich-like AuNP/analyte/AuNP structure. As a result, intensities of the Raman scattering peaks of wax/AuNPs-methyl parathion/AuNPs were significantly increased compared to that of wax/AuNPs-methyl parathion (green line in Figure 5A). Similar observation was made for melamine as shown in Figure 5B. The results demonstrate that the wax/AuNPs-analytes/AuNPs detection strategy developed here can potentially detect methyl parathion and melamine with high sensitivity.

### Sensitivity of SERS sensor system toward targets

**Figure 6:**
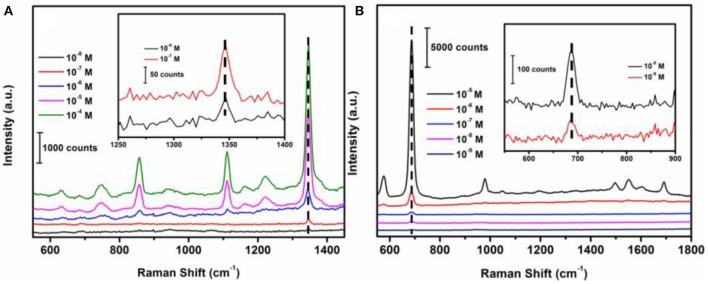
Raman scattering spectra of wax/AuNPs-methyl parathion/AuNPs at different methyl parathion concentrations varied from 10^−8^ M to 10^−4^ M **(A)** and wax/AuNPs-melamine/AuNPs at different melamine concentrations varied from 10^−9^ M to 10^−5^ M **(B)**.

In order to determine the sensitivity of the SERS sensor, methyl parathion in ethanol and melamine in water were prepared at a range of concentrations from 1 × 10^−4^ M to 1 × 10^−8^ M. The strongest peaks at 1346.6 and 687.4 cm^−1^ were selected for concentration-dependence SERS studies of methyl parathion and melamine, respectively. Figure 6A depicted the Raman spectra of methyl parathion with concentrations varying from 1 × 10^−4^ M to 1 × 10^−8^ M. It was clear that the Raman intensities significantly decreased with decreased concentration of methyl parathion. Inset of Figure 6A represents the Raman spectra at low methyl parathion concentrations, showing a clearly distinguishable peak at 1346.6 cm^−1^ even at methyl parathion concentration of 10 nM. The result demonstrated that the wax/AuNPs-methyl parathion/AuNPs displays high sensitivity with detection limit down to nanomolar level (S/N > 3) for methyl parathion. Similar results depicted in Figure 6B were obtained for melamine. Raman scattering spectra intensities obviously increased with increase of melamine concentrations. The Raman scattering spectra at low concentrations melamine were shown in inset of Figure 6B. A distinct peak at 687.4 cm^−1^ can be obtained at low melamine concentration of 1 × 10^−9^ M, which demonstrates that the wax/AuNPs-melamine/AuNPs can detect melamine at sub-nanomolar level (S/N >3). These results proved that the wax/AuNPs-analyte/AuNPs based SERS strategy is highly sensitive toward both methyl parathion and melamine.

### Reproducibility and practicability

**Figure 7:**
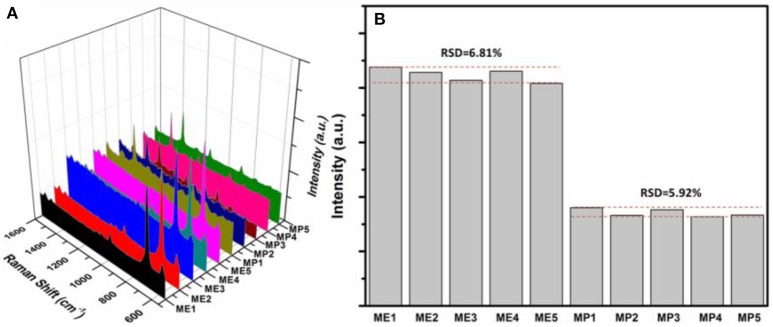
**(A)** Raman scattering spectra of 5 wax/AuNPs-melamine/AuNPs samples and 5 wax/AuNPs-methyl parathion/AuNPs samples prepared at melamine and methyl parathion concentrations of 1 × 10^−6^ M. **(B)** Plot of the absolute Raman intensities at 687.4 cm^−1^ for melamine and 1346.6 cm^−1^ for methyl parathion from five different samples, respectively.

Excellent hydrophobicity of wax-coated surface favors the uniformity of the distribution of AuNPs-analyte/AuNPs on the substrate after drying, thus resulting in better reproducibility in SERS sensing. Also the use of a fiber-coupled portable Raman spectrometer is beneficial to the reproducibility, because the optical fiber collects signal from a much larger area, compared to the desktop Raman microscope where the laser beam is highly focused. As shown in Figure 7A, Raman scattering spectra of quintuplicate methyl parathion and melamine samples at an analyte concentration of 1 × 10^−6^ M were recorded. Similar peak intensities were obtained for both methyl parathion and melamine in all five samples. Figure 7B shows the absolute intensities for melamine and methyl parathion after subtraction of the background signal. As shown, the absolute intensities obtained were almost constant for methyl parathion and melamine, with a small relative standard deviation (RSD) of 6.81 and 5.92%, respectively, demonstrating a satisfactory reproducibility in SERS-based detection toward both analytes.

To study the feasibility of wax/AuNPs-analyte/AuNPs based SERS platform in real applications, lake water spiked with methyl parathion and milk spiked with melamine were prepared with desired analyte concentrations (1 × 10^−5^ M, 1 × 10^−6^ M, and 1 × 10^−7^ M). After sample processing as described in previous experimental section, the Raman spectra of the samples were collected. The Raman peak intensities at 1346.6 and 687.4 cm^−1^ were used to calculate the recovery for methyl parathion and melamine, respectively. As shown in Table 2, recoveries of methyl parathion in spiked lake water ranges from 97.3 to 114.2% with a relative standard deviation <9.11%, suggesting excellent recoveries for methyl parathion detection in lake water samples. However, low recoveries of melamine ranging from 59.3 to 68.6% were obtained in spiked milk samples. The low recoveries are likely caused by the sample treatment/melamine recovery procedure before Raman detection. In our study, to recover melamine in milk, the milk proteins and other macromolecules were denatured/precipitated using methanol, followed by centrifugation for removal. Denatured/precipitated proteins/macromolecules are naturally absorbents, and thus can adsorb small molecules like melamine, resulting in the loss of melamine in supernatant for the SERS detection. In spite of the relatively lower recovery, the maximum of melamine content of 2.5 ppm (20 μM) in milk regulated by many international agencies can be easily detected (Wu et al., 2012; Trapiella-Alfonso et al., 2013). These results have demonstrated that the wax/AuNPs-analyte/AuNPs based SERS sensing strategy holds great potential in real applications for food safety and environment protection.

**Table 2:** Recovery studies of methyl parathion in spiked lake water samples and melamine in spiked milks samples.

**Spiked samples**	**Added amount (μM)**	**Recoveries (%)**	**RSD (%)**
Lake water spiked with MP	10	98.6	9.03
	1	114.2	9.11
	0.1	97.3	6.58
Milk spiked with Me	10	68.6	11.09
	1	59.3	9.11
	0.1	66.4	5.27

## Conclusions

In summary, we have presented a versatile, rapid and low-cost SERS sensing platform enabled by AuNPs-analyte/AuNPs double-decker structure on wax-coated hydrophobic surface using a portable Raman spectrometer. The method has been successfully applied for sensitive and selective detection of methyl parathion and melamine. Wax coating provides a universal, simple and low-cost method to produce highly hydrophobic substrates, which can enhance the sensitivity and reproducibility of SERS detection of analytes in water or polar solvents. In addition, by coating another layer of AuNPs on AuNPs-analyte to form double-decker structure, the sensitivity of the method is further improved significantly due to the formation of more “hot spots.” Remarkable recovery for methyl parathion spiked into lake water and acceptable recovery for melamine spiked into milk have been obtained, indicating good applicability and feasibility of the developed sensor platform for real applications. All these features demonstrated that the wax/AuNPs-analyte/AuNPs based SERS sensing strategy can potentially be used in sensing trace amount of chemicals for food and environmental safety applications.

## Author contributions

HW conducted the SERS experiments and initiated the writing of the manuscript. YiL and JZ synthesized AuNPs. YH and QD discussed the results and processed data. CH and JZ revised the manuscript. DH and YuL designed the experiment, managed the project, and finalized the manuscript.

### Conflict of interest statement

The authors declare that the research was conducted in the absence of any commercial or financial relationships that could be construed as a potential conflict of interest.

## References

[B1] Albrecht M. G. Creighton J. A. (1977). ChemInform Abstract: ANOMALOUSLY INTENSE RAMAN SPECTRA OF PYRIDINE AT A SILVER ELECTRODE. Cheminform 8, 5215–5217. 10.1002/chin.197901013

[B2] Alsammarraie F. K. Lin M. (2017). Using standing gold nanorod arrays as surface-enhanced raman spectroscopy (SERS) substrates for detection of carbaryl residues in fruit juice and milk. J. Agric. Food Chem. 65, 666–674. 10.1021/acs.jafc.6b04774 28080039

[B3] Camden J. P. Dieringer J. A. Wang Y. Masiello D. J. Marks L. D. Schatz G. C.. (2008). Probing the structure of single-molecule surface-enhanced Raman scattering hot spots. J. Am. Chem. Soc. 130, 12616–12617. 10.1021/ja8051427 18761451

[B4] Cao X. Hong S. Jiang Z. She Y. Wang S. Zhang C.. (2017). SERS-active metal-organic frameworks with embedded gold nanoparticles. Analyst 142, 2640–2647. 10.1039/C7AN00534B 28612075

[B5] Cheung M. Lee W. W. Y. Mccracken J. N. Larmour I. A. Brennan S. Bell S. E. J. (2016). Raman analysis of dilute aqueous samples by localized evaporation of submicroliter droplets on the tips of superhydrophobic copper wires. Anal. Chem. 88, 4541–4547. 10.1021/acs.analchem.6b00563 27031750

[B6] Fleischmann M. Hendra P. J. Mcquillan A. J. (1974). Raman spectra of pyridine adsorbed at a silver electrode. Chem. Phys. Lett. 26, 163–166. 10.1016/0009-2614(74)85388-1

[B7] Fortuni B. Fujita Y. Ricci M. Inose T. Aubert R. Lu G.. (2017). A novel method for *in situ* synthesis of SERS-active gold nanostars on polydimethylsiloxane film. Chem. Commun. 53, 5121–5124. 10.1039/C7CC01776F 28435951

[B8] Freeman R. G. Grabar K. C. Allison K. J. Bright R. M. Davis J. A. Guthrie A. P.. (1995). Self-assembled metal colloid monolayers: an approach to SERS substrates. Science 267, 1629–1632. 10.1126/science.267.5204.1629 17808180

[B9] Frens G. (1973). Controlled nucleation for the regulation of the particle size in monodisperse gold suspensions. Nature 241, 20–22.

[B10] Giovannozzi A. M. Rolle F. Sega M. Abete M. C. Marchis D. Rossi A. M. (2014). Rapid and sensitive detection of melamine in milk with gold nanoparticles by Surface Enhanced Raman Scattering. Food Chem. 159, 250–256. 10.1016/j.foodchem.2014.03.013 24767052

[B11] Haiss W. Thanh N. T. Aveyard J. Fernig D. G. (2007). Determination of size and concentration of gold nanoparticles from UV-Vis spectra. Anal. Chem. 79, 4215–4221. 10.1021/ac0702084 17458937

[B12] He L. Shi J. Sun X. Lin M. Yu P. Li H. (2011). Gold coated zinc oxide nanonecklaces as a SERS substrate. J. Nanosci. Nanotechnol. 11, 3509–3515. 10.1166/jnn.2011.3736 21776731

[B13] Hogard M. L. Lunte C. E. Lunte S. M. (2017). Detection of reactive aldehyde biomarkers in biological samples using solid-phase extraction pre-concentration and liquid chromatography with fluorescence detection. Anal. Methods 9, 1848–1854. 10.1039/C6AY03327J

[B14] Hu Y. Feng S. Gao F. Li-Chan E. C. Y. Grant E. Lu X. (2015). Detection of melamine in milk using molecularly imprinted polymers-surface enhanced Raman spectroscopy. Food Chem. 176, 123–129. 10.1016/j.foodchem.2014.12.051 25624214

[B15] Ilkhani H. Hughes T. Li J. Zhong C. J. Hepel M. (2016). Nanostructured SERS-electrochemical biosensors for testing of anticancer drug interactions with DNA. Biosens. Bioelectron. 80, 257–264. 10.1016/j.bios.2016.01.068 26851584

[B16] Jayram N. D. Aishwarya D. Sonia S. Mangalaraj D. Kumar P. S. Rao G. M. (2016). Analysis on superhydrophobic silver decorated copper Oxide nanostructured thin films for SERS studies. J. Colloid Interface Sci. 477, 209–219. 10.1016/j.jcis.2016.05.051 27294970

[B17] Jeanmaire D. L. Duyne R. P. V. (1977). Surface raman spectroelectrochemistry: Part I. Heterocyclic, aromatic, and aliphatic amines adsorbed on the anodized silver electrode. J. Electroanal. Chem. Interfac. Electrochem. 84, 1–20.

[B18] Jiang C. Ma X. Xue M. Lian H.-Z. (2017). Application of thermoresponsive hydrogel/gold nanorods composites in the detection of diquat. Talanta 174, 192–197. 10.1016/j.talanta.2017.06.010 28738567

[B19] Kim A. Barcelo S. J. Williams R. S. Li Z. (2012). Melamine sensing in milk products by using Surface Enhanced Raman Scattering. Anal. Chem. 84, 9303–9309. 10.1021/ac302025q 23043560

[B20] Kleinman S. L. Frontiera R. R. Henry A.-I. Dieringer J. A. Van Duyne R. P. (2013). Creating, characterizing, and controlling chemistry with SERS hot spots. Phys. Chem. Chem. Phys. 15, 21–36. 10.1039/C2CP42598J 23042160

[B21] Kubackova J. Fabriciova G. Miskovsky P. Jancura D. Sanchez-Cortes S. (2015). Sensitive Surface-Enhanced Raman Spectroscopy (SERS) detection of organochlorine pesticides by alkyl dithiol-functionalized metal nanoparticles-induced plasmonic hot spots. Anal. Chem. 87, 663–669. 10.1021/ac503672f 25494815

[B22] Kurzatkowska K. Santiago T. Hepel M. (2017). Plasmonic nanocarrier grid-enhanced Raman sensor for studies of anticancer drug delivery. Biosens. Bioelectron. 91, 780–787. 10.1016/j.bios.2017.01.049 28142123

[B23] Lee S. Choi J. Chen L. Park B. Kyong J. B. Seong G. H.. (2007). Fast and sensitive trace analysis of malachite green using a surface-enhanced Raman microfluidic sensor. Anal. Chim. Acta 590, 139–144. 10.1016/j.aca.2007.03.049 17448337

[B24] Li J. Chen L. Lou T. Wang Y. (2011). Highly sensitive SERS detection of As3+ ions in aqueous media using glutathione functionalized silver nanoparticles. ACS Appl. Mater. Interfaces 3:3936. 10.1021/am200810x 21916441

[B25] Li J. J. An H. Q. Zhu J. Zhao J. W. (2015). Improve the Surface Enhanced Raman Scattering of gold nanorods decorated graphene oxide: the effect of CTAB on the electronic transition. Appl. Surf. Sci. 347, 856–860. 10.1016/j.apsusc.2015.04.194

[B26] Li X. Lee H. K. Phang I. Y. Lee C. K. Ling X. Y. (2014). Superhydrophobic-oleophobic Ag nanowire platform: an analyte-concentrating and quantitative aqueous and organic toxin surface-enhanced Raman scattering sensor. Anal. Chem. 86, 10437–10444. 10.1021/ac502955w 25230236

[B27] Li Z. Meng G. Huang Q. Hu X. He X. Tang H.. (2015). Ag nanoparticle-grafted PAN-nanohump array films with 3D high-density hot spots as flexible and reliable SERS substrates. Small 11, 5452–5459. 10.1002/smll.201501505 26313309

[B28] Lim D. K. Jeon K. S. Kim H. M. Nam J. M. Suh Y. D. (2010). Nanogap-engineerable Raman-active nanodumbbells for single-molecule detection. Nat. Mater. 9, 60–67. 10.1038/nmat2596 20010829

[B29] Liu H. L. Cao J. Hanif S. Yuan C. Pang J. Levicky R.. (2017). Size-Controllable Gold Nanopores with High SERS Activity. Anal. Chem. 89, 10407–10413. 10.1021/acs.analchem.7b02410 28853540

[B30] Lou T. Wang Y. Li J. Peng H. Xiong H. Chen L. (2011). Rapid detection of melamine with 4-mercaptopyridine-modified gold nanoparticles by surface-enhanced Raman scattering. Anal. Bioanal. Chem. 401, 333–338. 10.1007/s00216-011-5067-3 21573845

[B31] Mircescu N. E. Oltean M. Chiş V. Leopold N. (2012). FTIR, FT-Raman, SERS and DFT study on melamine. Vib. Spectrosc. 62, 165–171. 10.1016/j.vibspec.2012.04.008

[B32] Nguyen T. H. D. Zhang Z. Mustapha A. Li H. Lin M. (2014). Use of graphene and gold nanorods as substrates for the detection of pesticides by Surface Enhanced Raman Spectroscopy. J. Agric. Food Chem. 62, 10445–10451. 10.1021/jf5036417 25317673

[B33] Özyürek M. Baki S. Gungor N. Celik S. E. Guclu K. Apak R. (2012). Determination of biothiols by a novel on-line HPLC-DTNB assay with post-column detection. Anal. Chim. Acta 750, 173–181. 10.1016/j.aca.2012.03.056 23062438

[B34] Peng B. Li G. Li D. Dodson S. Zhang Q. Zhang J.. (2013). Vertically aligned gold nanorod monolayer on arbitrary substrates: self-assembly and femtomolar detection of food contaminants. ACS Nano 7, 5993–6000. 10.1021/nn401685p 23790104

[B35] Rajapandiyan P. Tang W.-L. Yang J. (2015). Rapid detection of melamine in milk liquid and powder by surface-enhanced Raman scattering substrate array. Food Control 56, 155–160. 10.1016/j.foodcont.2015.03.028

[B36] Schwartzberg A. M. Grant C. D. Wolcott A. Talley C. E. Huser T. R. Bogomolni R. (2004). Unique gold nanoparticle aggregates as a highly active Surface-Enhanced Raman Scattering Substrate. J. Phys. Chem. B 108, 19191–19197. 10.1021/jp048430p

[B37] Tan M. J. Hong Z.-Y. Chang M.-H. Liu C.-C. Cheng H.-F. Loh X. J.. (2017). Metal carbonyl-gold nanoparticle conjugates for highly sensitive SERS detection of organophosphorus pesticides. Biosens. Bioelectron. 96, 167–172. 10.1016/j.bios.2017.05.005 28494368

[B38] Tian X. D. Liu B. J. Li J. F. Yang Z. L. Ren B. Tian Z. Q. (2013). SHINERS and plasmonic properties of Au Core SiO2 shell nanoparticles with optimal core size and shell thickness. J. Raman Spectrosc. 4, 994–998. 10.1002/jrs.4317

[B39] Trapiella-Alfonso L. Costa-Fernandez J. M. Pereiro R. Sanz-Medel A. (2013). Synthesis and characterization of hapten-quantum dots bioconjugates: application to development of a melamine fluorescent immunoassay. Talanta 106, 243–248. 10.1016/j.talanta.2013.01.027 23598123

[B40] Vesely P. Lusk L. Basarova G. Seabrooks J. Ryder D. (2003). Analysis of aldehydes in beer using solid-phase microextraction with on-fiber derivatization and gas chromatography/mass spectrometry. J. Agric. Food Chem. 51, 6941–6944. 10.1021/jf034410t 14611150

[B41] Wallace R. A. Charlton J. J. Kirchner T. B. Lavrik N. V. Datskos P. G. Sepaniak M. J. (2014). Superhydrophobic analyte concentration utilizing colloid-pillar array SERS substrates. Anal. Chem. 86, 11819–11825. 10.1021/ac5033947 25368983

[B42] Wang Y. Wang H. Wang Y. Shen Y. Xu S. Xu W. (2016). Plasmon-driven dynamic response of a hierarchically structural silver-decorated nanorod array for sub-10 nm nanogaps. ACS Appl. Mater. Interfaces 8, 15623–15629. 10.1021/acsami.6b04173 27250862

[B43] Wang Y. Yan B. Chen L. (2013). SERS tags: novel optical nanoprobes for bioanalysis. Chem. Rev. 113, 1391–1428. 10.1021/cr300120g 23273312

[B44] Wu B. Wang Z. Zhao D. Lu X. (2012). A novel molecularly imprinted impedimetric sensor for melamine determination. Talanta 101, 374–381. 10.1016/j.talanta.2012.09.044 23158337

[B45] Yu Z. Park Y. Chen L. Zhao B. Jung Y. M. Cong Q. (2015). Preparation of a superhydrophobic and peroxidase-like activity array chip for H2O2 sensing by Surface-Enhanced Raman Scattering. ACS Appl. Mater. Interfaces 7, 23472–23480. 10.1021/acsami.5b08643 26437325

[B46] Zhang C. You E. Jin Q. Yuan Y. Xu M. Ding S.. (2017). Observing the dynamic “hot spots” on two-dimensional Au nanoparticles monolayer film. Chem. Commun. 53, 6788–6791. 10.1039/C7CC03020G 28597893

[B47] Zhou Q. Meng G. Wu N. Zhou N. Chen B. Li F. (2016). Dipping into a drink: Basil-seed supported silver nanoparticles as surface-enhanced Raman scattering substrates for toxic molecule detection. Sensors Actuat. B Chem. 223, 447–452. 10.1016/j.snb.2015.09.115

